# FocusDet: an efficient object detector for small object

**DOI:** 10.1038/s41598-024-61136-w

**Published:** 2024-05-10

**Authors:** Yanli Shi, Yi Jia, Xianhe Zhang

**Affiliations:** College of Information and Control Engineering, Jilin Institute of Chemical Technology, Jilin, 132000 China

**Keywords:** Engineering, Mathematics and computing

## Abstract

The object scale of a small object scene changes greatly, and the object is easily disturbed by a complex background. Generic object detectors do not perform well on small object detection tasks. In this paper, we focus on small object detection based on FocusDet. FocusDet refers to the small object detector proposed in this paper. It consists of three parts: backbone, feature fusion structure, and detection head. STCF-EANet was used as the backbone for feature extraction, the Bottom Focus-PAN for feature fusion, and the detection head for object localization and recognition.To maintain sufficient global context information and extract multi-scale features, the STCF-EANet network backbone is used as the feature extraction network.PAN is a feature fusion module used in general object detectors. It is used to perform feature fusion on the extracted feature maps to supplement feature information.In the feature fusion network, FocusDet uses Bottom Focus-PAN to capture a wider range of locations and lower-level feature information of small objects.SIOU-SoftNMS is the proposed algorithm for removing redundant prediction boxes in the post-processing stage. SIOU multi-dimension accurately locates the prediction box, and SoftNMS uses the Gaussian algorithm to remove redundant prediction boxes. FocusDet uses SIOU-SoftNMS to address the missed detection problem common in dense tiny objects.The VisDrone2021-DET and CCTSDB2021 object detection datasets are used as benchmarks, and tests are carried out on VisDrone2021-det-test-dev and CCTSDB-val datasets. Experimental results show that FocusDet improves mAP@.5% from 33.6% to 46.7% on the VisDrone dataset. mAP@.5% on the CCTSDB2021 dataset is improved from 81.6% to 87.8%. It is shown that the model has good performance for small object detection, and the research is innovative.

## Introduction

General object detectors have been developed and matured, but as more application scenarios are developed, the application of small object detection becomes more and more widespread. The accuracy of general object detectors is insufficient in detecting small objects. Detecting small objects holds significant importance in UAV aerial photography and vehicle autonomous driving systems. More accurate detection of small objects can make the system more robust and feasible decisions. In this study, a “small object” refers to an object occupying a small pixel area in the input image with a resolution of less than 32 pixels $\times$32 pixels. Several object detectors for small object detection have been proposed in recent years such as UIU-Net^1^. QueryDet^2^. DFPN^3^, GFL V1^4^. However, they are time-consuming and have high computational complexity. Therefore, it is not suitable for real-time detection of UAV aerial photography and vehicle automatic driving system.

With its excellent detection efficiency, the one-stage object detector YOLOv5 has been utilized for general object detection. However, further design is required to handle small object detection tasks. The size of small objects varies significantly and they contain a lot of complex background information. Following the features of the backbone network have been extracted. Semantic information about small items is lost. It is challenging to concentrate on their context-related information. In the Neck structure, YOLOv5 uses the PAN structure to enrich the feature map details. However, the image resolution decreases as the depth of the network increases.The lack of small object features leads to poor detection effect. The dense objects in the small object dataset are also a key point that affects detection performance. Post-processing using NMS is not suitable for processing dense small objects. During Non-maximum suppression, if an object appears in the overlapping threshold, it is discarded. In dense scenarios this can result in severe missed detections, leading to a decrease in average accuracy.

After comparison with a variety of classical object detectors.YOLOv5s is chosen as the infrastructure to propose the small object detector FocusDet. The network’s general organizational structure includes three parts: The backbone extracts image features to generate a feature map. Neck fuses feature maps of different depths.The Head performs position and category detection on the fused feature maps. FocusDet makes the following contributions to solving the precision problem:

(i) Efficient Small Object Detector FocusDet. There are small objects, dense objects, and objects with large-scale differences in images taken from complex scenes such as drones and underwater. To address these challenges, we propose FocusDet. High precision detection is achieved with low parameter numbers. It has good generalization ability. The performance of small object detection is significantly enhanced in complex scenes.
(ii) Strengthen the feature extraction network for small objects. In the process of feature extraction, small objects are prone to feature loss in the convolution process. As a result, missing detection and false detection occur in the detection phase. To address this challenge, we design efficient Enhancing Aggregation Networks with Small Target Context Features. The Locally enhanced Position Encoding Attention Module in the network is used to efficiently select small object features. The Space-to-depth module performs feature enhancement on small object features. It retains the richness and integrity of small object features to a great extent. It effectively fights the information loss caused by small objects in convolution.
(iii) We design the Bottom Focus-PAN for the feature loss phenomenon of deep small objects. This module effectively uses shallow features to fuse with deep features. Not only the large object features are preserved, but also the small object features are complemented. This provides an effective solution for the lack of deep feature information on small objects and further improves the detection accuracy of small objects.
(iv) Repeated detection and missed detection often occur in dense small object detection. To this end, the SIOU-SoftNMS module is proposed. SIOU is used to accurately locate the object box in multiple dimensions. SoftNMS^5^ is used to suppress the redundant object boxes. Without increasing the number of parameters, the detection effect of dense objects is effectively improved.

## Related work

### General object detection

The first two-stage object detection model RCNN^6^ was proposed. By generating a large number of candidate regions, these regions are fed into the CNN model for feature extraction. It will use SVM to classify the feature maps. Finally, the position of the candidate box is corrected. Fast R-CNN^7^ is trained by combining classification loss with bounding box regression loss and also uses the Softmax classifier instead of the SVM classifier. However, all the above algorithms use Selective Search to obtain candidate regions. The computational overhead is large and this method is more time-consuming. Faster R-CNN uses a Region Proposal Network (RPN) and combines the Anchor mechanism to generate candidate boxes, which improves the speed of the model. Being the first algorithm that comes closest to real-time object detection. The Faster R-CNN^8^ method is currently the mainstream object detection method, but the speed can not meet the real-time requirements. The model predicts only the last layer feature map. This is not conducive to small object detection with limited information.

Compared with the two-stage detection algorithm, single-stage object detection is more suitable for small object scenes that require real-time detection. Joseph Redmon et al.^9^proposed YOLO (You Only Look Once) in 2016, which treated the detection problem as a regression problem and pioneered one-stage object detection. It was followed the next year by Joseph Redmon et al .YOLO9000^10^ is a model that can detect more than 9000 different kinds of objects. Although YOLO runs fast, it has a low recall rate and poor object detection effect. Yolov3^11^ uses a deeper DarkNet-53 to extract image features. The model can detect feature maps of multiple scales, which improves the performance of object detection. The latest Yolov8 in 2023, the updated c2f module has a better effect on common object feature extraction. The Yolo series has achieved excellent results in general object detection. However, in the face of small object scenes, the feature details of small objects are seriously lost in the feature extraction process. The feature fusion structure is insufficient to utilize the features of small objects. This leads to false detection and missing detection in small object detection. In this paper, for small object detection, we use the Space to depth module to strengthen small object features and AttentionLepeC3 module selects features. Meanwhile, a new feature fusion structure is designed to fuse the features of small objects. The accuracy of small object detection is greatly improved.

### Small object detection

Small object detection applications are essential in UAV platforms and autonomous driving application scenarios. General object detectors have many problems in handling small object detection tasks. Such as low recall and slow detection speed. The following are the primary causes: The small object occupies a small area, which is susceptible to background interference in a complex background. The small size of the object has low resolution. Small object features are lost during convolutional computation. Small objects often appear densely and are heavily obscured. Small object detectors need to be targeted and designed according to the characteristics of small object datasets. Some researchers have already focused on this aspect and proposed feasible solutions.

Improving the resolution of the images is an effective and direct method. High-resolution images enable Backbone to effectively extract small object features. Li et al.^12^proposed a Perceptual Generative Adversarial Network, which is specialized for small object detection. The generator of this network maps the small object features to those similar to the large object, which enhances the feature representation of the small object. However, the Generative Adversarial Network has a high complexity and is difficult to train, requiring a special training strategy. To retain the feature loss of reducing small objects, this paper uses a better small object feature extraction network STCF-EANet to solve this problem. The Space to depth module performs feature enhancement and the AttentionLePE module performs feature selection. Small object features are well preserved.

To solve the problem of insufficient deep feature semantic information, the common methods use multi-scale learning for feature fusion. In 2017, Lin et al.^13^ proposed Feature Pyramid Networks. The method of upsampling the low-resolution deep feature maps and then fusing the shallow feature maps improves the problem of insufficient information on small objects in the deep features. However, this method has a good effect on general object detection data, and small object data still has insufficient features. In recent years, some researchers have designed small object detection heads^14^ to directly detect shallow feature maps. The rich features of shallow small objects are used to improve the detection effect. However, due to the addition of shallow detection heads, the amount of calculation becomes larger and the computational complexity is greatly increased. In this paper, the Bottom Focus-PAN is proposed. The computation and computational complexity are not high. And it makes full use of the underlying feature information for fusion. To make up for deep losses small object features.

In the face of dense small object scenes, common algorithms are prone to miss detection. To solve the miss detection of small objects, Law et al.^15^ proposed a CornerNet algorithm based on corner detection in 2018. The algorithm first predicts the top left and bottom right corners of each object. The second step matches the top left corner and bottom right corner of the same object based on the detected corner embedding vector. Finally, the position of the corner is adjusted by the offset to obtain the object bounding box. However, CornerNet tends to ignore the internal information of the object. To improve this problem, duan et al.^16^ proposed a CenterNet algorithm to eliminate false bounding boxes using central key points. However, the repeated detection phenomenon of this algorithm is serious. SIOU-SoftNMS method is proposed in this paper. The anchor box is located in multiple dimensions, and a new elimination mechanism is proposed for the wrong anchor box. It greatly alleviates the problem of dense object omission detection.

Based on the advantages and disadvantages of the current small object research results, new feasible solutions are explored. We present FocusDet small object detector. It is better at small object detection.

## Proposed method

### Overall structure of FocusDet

**Figure 1:**
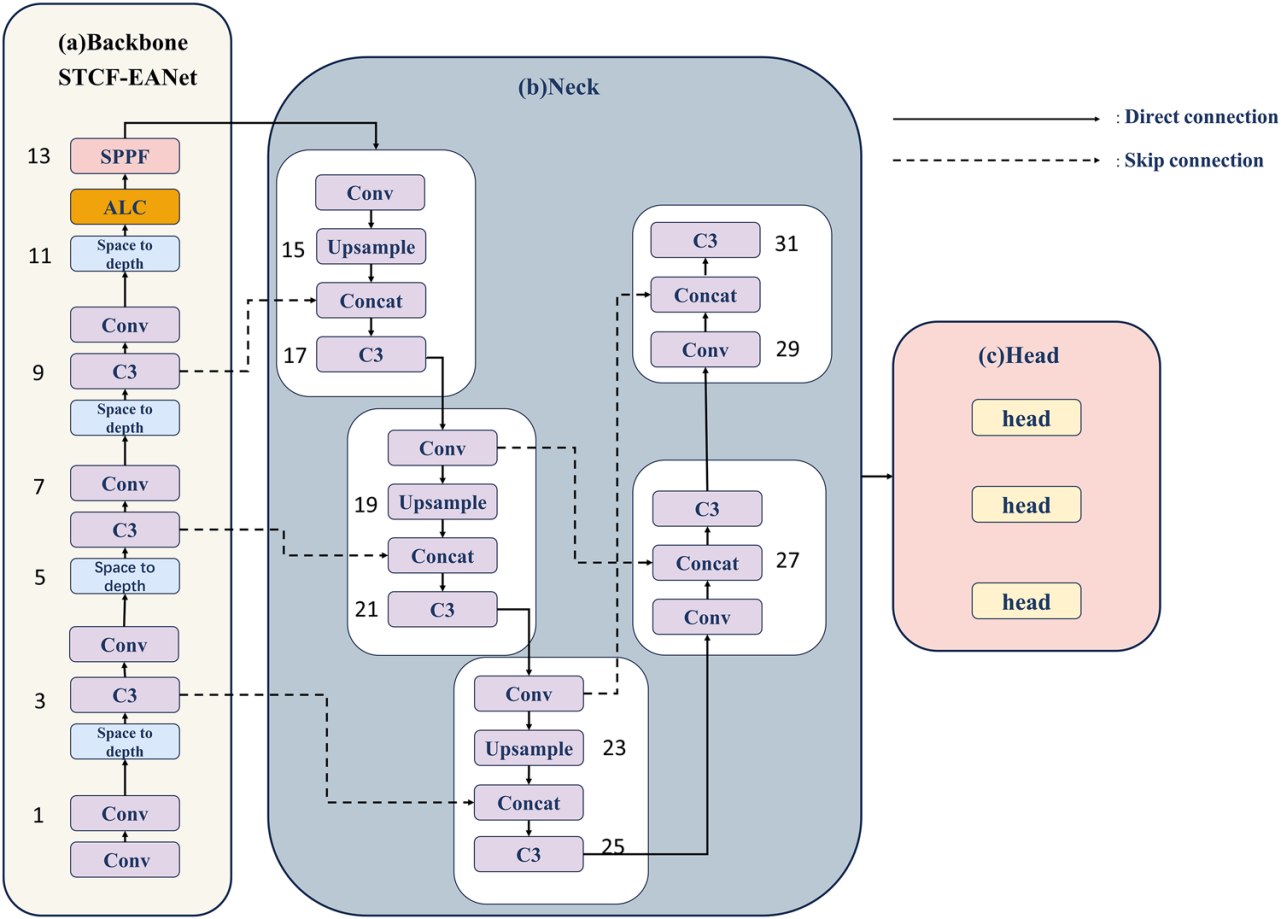
Structure of FocusDet.

After the comparison of multiple object detectors, yolov5 was developed and matured. It is better at handling small object tasks and is suitable to be chosen as a benchmark model. An object detector for the small object detector FocusDet is proposed.By improving the backbone^11^, feature aggregation network, and post-processing non-maximum suppression. In the backbone network, Enhancing Aggregation Networks with Small Target Context Features (STCF-EANet) is proposed. Backbone adds a step-free convolution module Space-to-depth and a locally enhanced position-encoding attention module AttentionLepeC3 (ALC)^17^. The non-strided convolution module makes the network retain more small object details. ALC enables networks to better capture small object features. In the feature fusion network, Bottom Focus-PAN is designed to solve the problem of insufficient feature information for small objects. The small object feature details in the deep feature map are supplemented. During post-processing, the existing methods make it easy to generate low-quality redundant detection boxes. SIOU-SoftNMS is designed to improve the common problem of dense clusters accompanying small objects and low detection recall rate caused by occlusion. The above improvements enhance FocusDet’s capacity to identify small objects. The structure of FocusDet is shown in Fig. 1.

### Enhancing aggregation networks with small target context features

**Figure 2:**
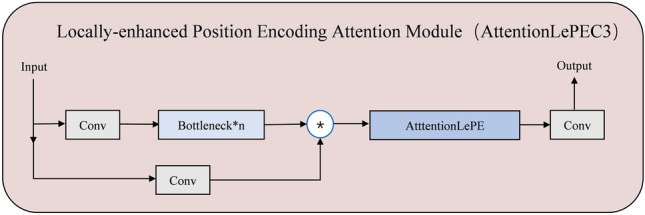
Structure of AttentionLePEC3.

The small object features are too small, the semantic information is insufficient. As a result, the backbone network makes it difficult to extract small object features. To address this issue, a small target context feature enhancement aggregation network is proposed. The small target context feature enhancement aggregation network STCF-EANet is integrated into the backbone^11^ by two modules. The two modules are the space-to-depth^18^ module and the locally enhanced position-encoded attention module AttentionLepeC3 (ALC).It makes the small object features more obvious. In the actual detection effect, the field of view is wider and the recognition accuracy is higher. The ALC structure is shown in Fig. 2.

### Locally-enhanced position encoding attention module

AttentionLepe refers to the Cswin Transformer’s LePE^17^ designed on Attention to enhance the local position information. Attention includes three parts: Q(query), K(key), and V(value). Firstly, Weight is obtained by calculating the degree of correlation between Q and each K. By calculating the correlation between Q and K, the importance degree of different K to the output is obtained.

1 $$\begin{aligned} f(Q,K_{i})=Q^{T}K \end{aligned}$$

Softmax function was used to normalize these weights.

2 $$\begin{aligned} a_i=soft\max \left( f(Q,K_i)\right) =\frac{\exp \left( f(Q,K_i)\right) }{\Sigma _j\exp \left( f(Q,K_j)\right) } \end{aligned}$$

Attention is obtained by the weighted sum of the weights and the corresponding key value.

3 $$\begin{aligned} Attention\left( Q,K,V\right) =\Sigma _{i}a_{i}V_{i} \end{aligned}$$

**Figure 3:**
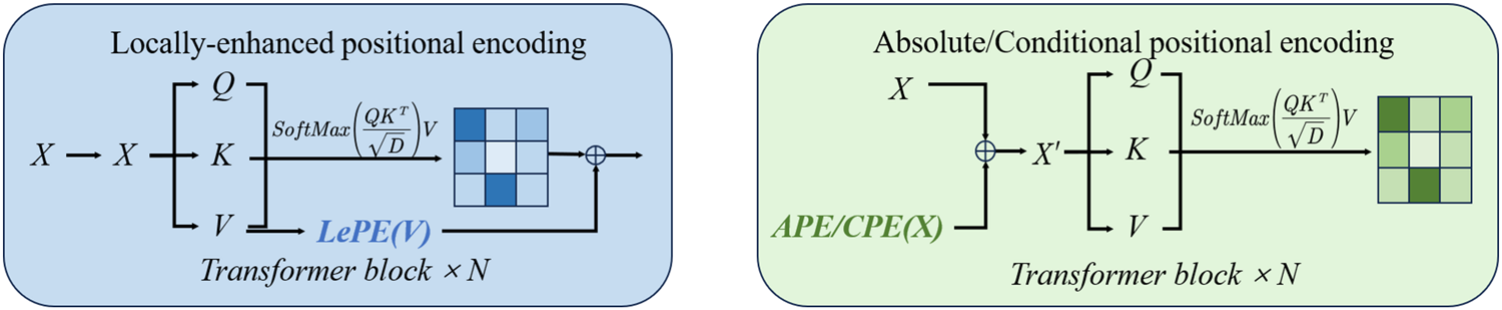
Comparison of the various positional encoding methods: LePE, APE, and CPE.

Positional information is immediately added to the input token of self-attention in positional encoding by APE (absolute positional encoding) and CPE (conditional positional encoding). Following that, it gets fed into the transformer block for the calculation of self-attention. APE and CPE act directly on the input and are for a specific size, so they are not suitable for images with different resolutions. Conversely, position encodings can be produced by RPE at any input resolution. Introducing a local inductive bias, LePE is incorporated into the self-attention branch as a parallel module. CSWinTransformer also uses a relative positional encoding (RPE), but it adds positional information to the calculation of attention. It considers imposing position information directly on the Value, and then adding the Value with position encoding and self-attention weighting together utilizing residual. APE and CPE are the position information introduced before feeding into the Transformer module, while RPE and LePE are operated in each Transformer module with higher flexibility and better effect. As shown in Fig. 3.SoftMax value is added by LePE, which operates directly on value. AttentionLePE is calculated as follows.

4 $$\begin{aligned} Attention(Q,K,V)=SoftMax\Big (QK^{T}/\sqrt{d}\Big )+DWConv(V) \end{aligned}$$

**Figure 4:**
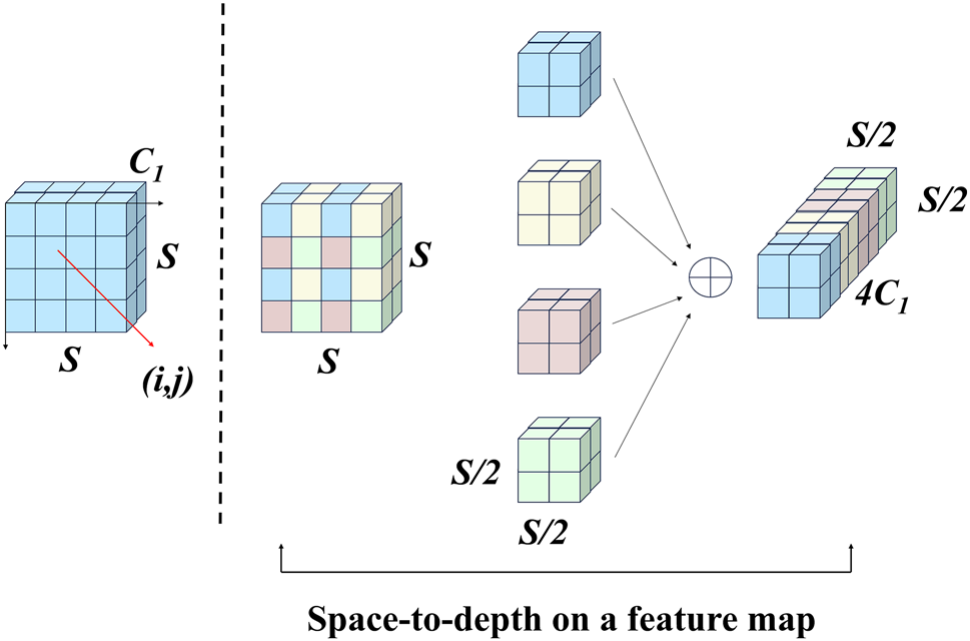
Space-to-depth processing.

### Space-to-depth module

A non-strided convolutional layer with a Space-to-depth^18^ (SPD)layer makes up Space-to-depth.$S\times S\times C_1$Size of the feature map $\text {X}$, slice out the subfeature map as:

5 $$\begin{aligned} \begin{aligned}f_{0,0}=X[0:S:scale,0:scale],f_{1,0}=X[1:S:scale,0:S:scale],...,\\ f_{scale-1,0}=X[scale-1{:}S{:}scale,0{:}S{:}scale];\\ f_{0,1}=X[0{:}S{:}scale,1{:}S{:}scale],f_{1,1},...,\\ f_{scale\text {\textit{ }}-1,1}=X[scale\text {\textit{ }}-1{:}S{:}scale,1{:}S{:}scale];\\ f_{0,scale\text {\textit{ }}-1}=X[0:S{:}scale,scale-1{:}S{:}scale],f_{1,scale{-1}},...,\\ f_{scale\text {\textit{ }}-1,scale\text {\textit{ }}-1}=X[scale-1:S{:}scale,scale-1{:}S{:}scale]\end{aligned}\end{aligned}$$

Generally speaking, given any feature map $\text {X}$, a sub-map $f_{x,y}$ is formed by all the entries $X_{(i,j)}$ that $\text {i+x}$ and are divisible by scale. Consequently,$\text {X}$ is downsampled by a scale factor in each sub-map. Figure 4 gives an example when scale = 2, where we obtain four sub-maps $f_{0,0},f_{1,0},f_{1,1},f_{0,1}$ each of which is of shape $(S/2,S/2,C_1)$ and downsamples $\text {X}$ by a factor of 2. Following the layer of SPD feature transformation, we add a non-strided convolution layer with $C_{2}$ filters where $C_2.\quad <\quad scale^2C_1$,And further transforms $X'\Big (\frac{S}{scale},\frac{S}{scale},scale^2C_1\Big )\rightarrow X''\Big (\frac{S}{scale},\frac{S}{scale},C_2\Big )$.As far as feasible, preserve all information related to discriminative features.

**Figure 5:**
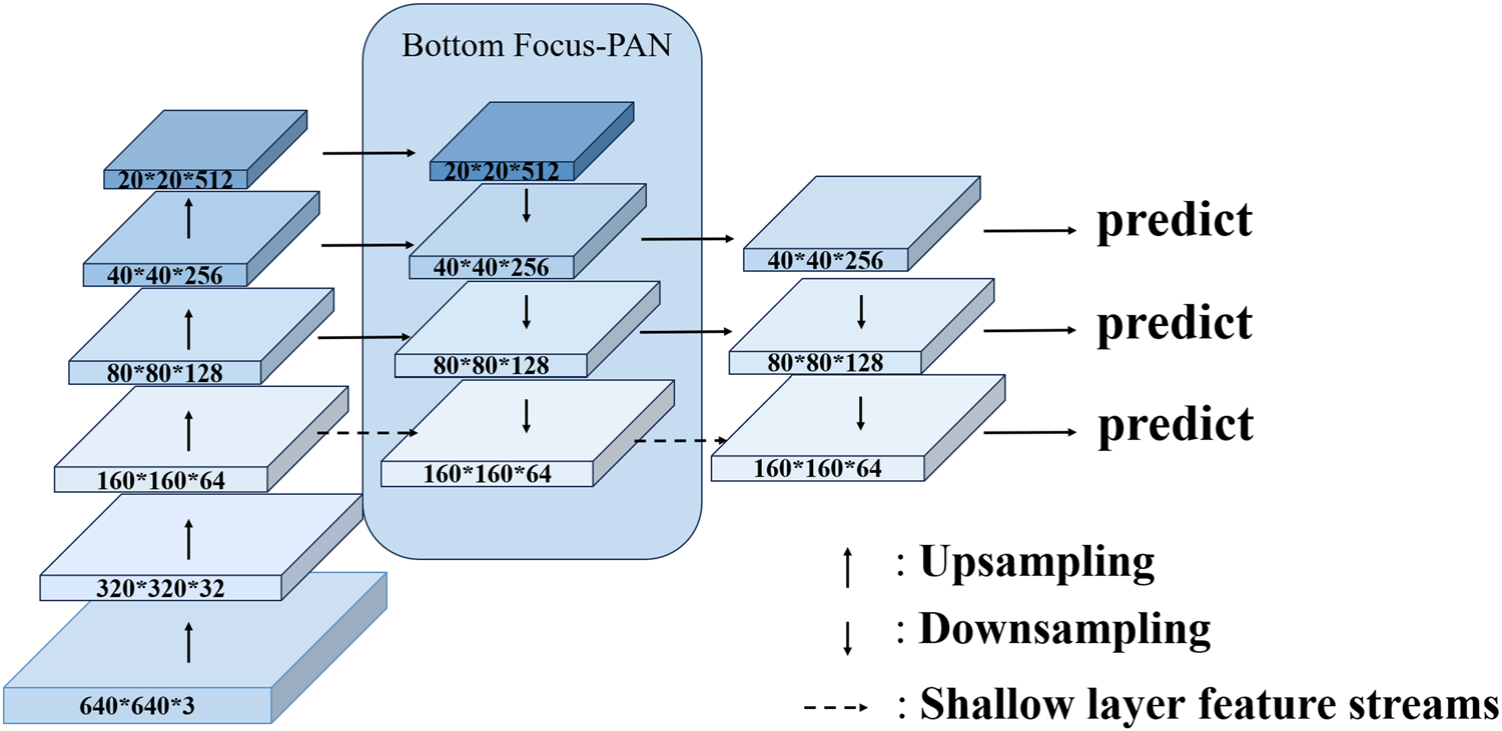
Bottom Focus-PAN structure.

### Bottom focus-PAN

Bottom Focus-PAN integrates contextual information and is a top-down structure that fuses feature maps from lower and higher layers. This is shown in Fig. 5. A More full utilization of the shallow feature map, which is richer in small object features. The structure can obtain 4 ×, 8 ×, and 16 × subsampled feature maps, and the input image pixels are 640*640, of which four times subsampled is 160*160 pixels. Low-resolution images lose details of object features. Bottom Focus-PAN makes full use of shallow feature maps for feature fusion to supplement rich small object feature details to the feature map. It improves the phenomenon of insufficient semantic information about objects and the inability to detect small objects in complex backgrounds.

**Figure 6:**
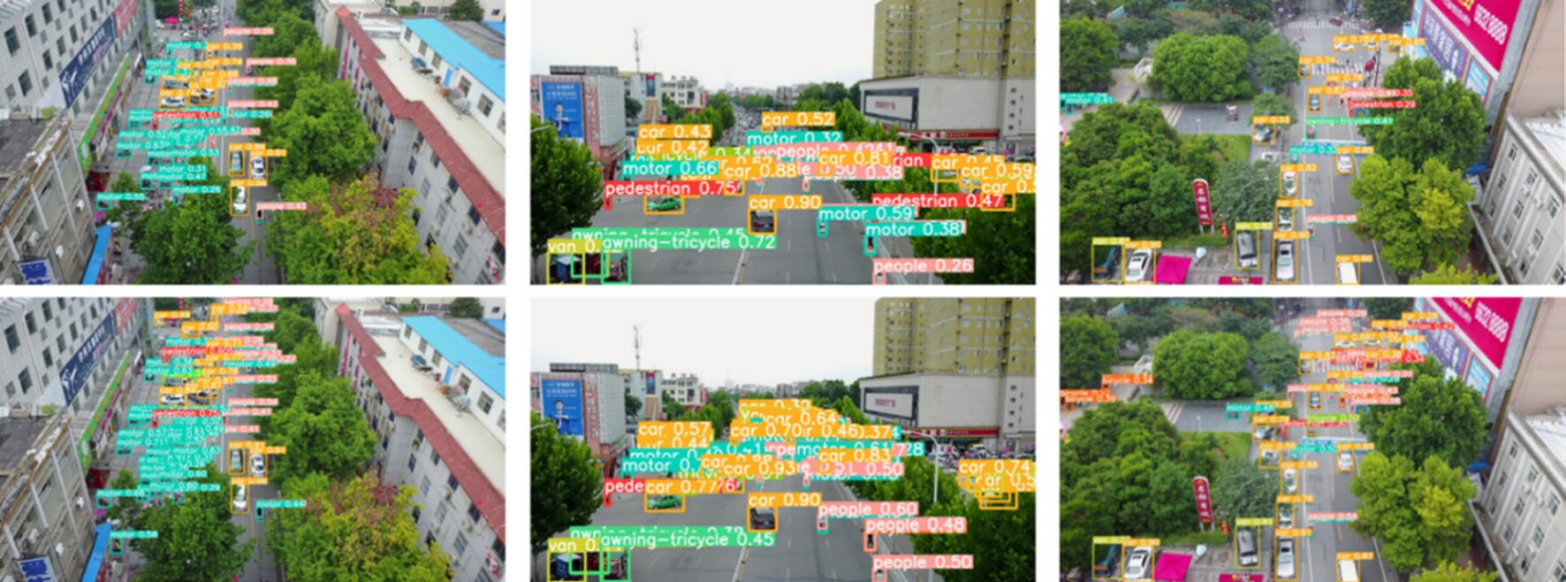
Comparison of the detection field of YOLOv5s (top) and Bottom Focus Pan (bottom).

Figure 6 illustrates the better handling of small objects by Bottom Focus-PAN compared to the original PAN.Bottom Foucs-PAN makes full use of 160*160*64 feature maps and fuses them with 80*80*128 upsampled feature maps. It supplements the feature details of small objects and retains the feature information of large objects. The detection effect is further improved. There are often scattered small objects on the edge of the image, which are easy to miss. After using Bottom Focus-PAN, The recognition of the small objects on the edge is seen. The small object in the upper and central regions of the frame is not recognized by YOLOv5s. Even so, With the Bottom Focus-PAN, FocusDet can handle the issue with effectiveness.

### SIOU-SoftNMS

The NMS algorithm has serious omissions when dealing with dense small object detections. When the two prediction boxes’ IOU exceeds the IOU threshold, the NMS algorithm directly removes the prediction box with less confidence. Replacing the NMS algorithm with SIOU-SoftNMS^5^ can better mitigate the dense small object omission phenomenon and better localize and predict the object without adding additional parameters. Prediction boxes below the confidence threshold are eliminated. To get the prediction box with the highest confidence, sort the boxes according to decreasing order of confidence. Set the IOU threshold, traverse all the prediction boxes, and if the IOU with the current highest confidence prediction box is greater than the IOU threshold, use the Gaussian method. as shown in Formula 6. The confidence of the prediction box is attenuated according to the degree of overlap. Instead of NMS removing the prediction box directly. Ultimately the accurate prediction box is left.

6 $$\begin{aligned} s_i=s_ie^{-\frac{IOU(\mathcal {M},b_i)^2}{\sigma }},\forall b_i\notin \mathcal {D} \end{aligned}$$

$IOU\left( M,b_i\right)$represents the IOU of the prediction box $\text {M}$ with the maximum confidence score concerning the $\text {i}$th prediction box$b_{i}$,$N_{t}$represents the threshold for repetition, and$S_{i}$represents the confidence score of the $\text {i}$th prediction box.

**Figure 7:**
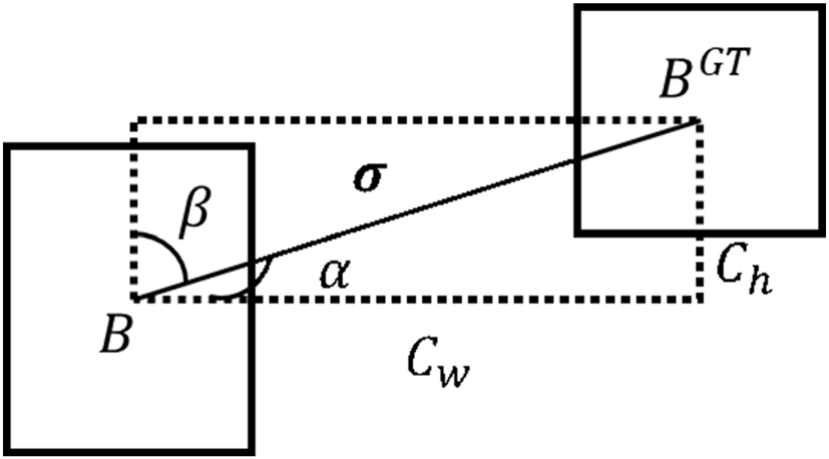
SIOU structure.

In the above approach, the IOU computation is performed in Soft-NMS using the SIOU^19^ computation method, which results in more accurate localization. Siou^19^ is calculated as follows:

7 $$\begin{aligned} SIOU=1-IOU+\frac{1}{2}(\cos t_{dis\tan ce}+\cos t_{shape})^{\leftrightarrow }\end{aligned}$$

Distance cost:

8 $$\begin{aligned} \begin{aligned}\Delta&=\Sigma _{t{\text {=}}=x,y}\left( 1-e^{-\gamma \rho _{t}}\right) =2-ex^{-\gamma \rho _{x}}-e^{-\gamma \rho _{y}{\leftarrow }}\\&\\ \rho _{x}&=\left( \frac{b_{c_x}^{g^t}-b_{c_x}}{W}\right) ^2,\rho _{y}{\text {=}}\left( \frac{b_{c_y}^{g^t}-b_{c_y}}{H}\right) ^2,\gamma =2-\Lambda ^{}\end{aligned}\end{aligned}$$

Angle cost:

9 $$\begin{aligned} \begin{aligned}\Lambda =&1-2\sin ^2\left( \arcsin \left( x\right) -\frac{\pi }{4}\right) \end{aligned}\end{aligned}$$

Shape cost:

10 $$\begin{aligned} \begin{aligned}\Omega =&(1-e^{-w_w})^\theta +(1-e^{-w_h})^{\theta _{}}\end{aligned}\end{aligned}$$

IOU cost:

11 $$\begin{aligned} IOU=\frac{|B\cap B^{GT}|}{|B\cup B^{GT}|}\end{aligned}$$

where$(w,h),(w^{gt},h^{gt})$denotes the width and height of the prediction box and the ground true box, respectively, and$(c_{w},c_{h})$is the width and height of the smallest outer rectangle of the ground truth box and the prediction box as shown in Fig. 7.

### Ethics declarations

There are no experiments on humans and animals involved in this study.

## Experiment

### Datasets and evaluation metrics

**Figure 8:**
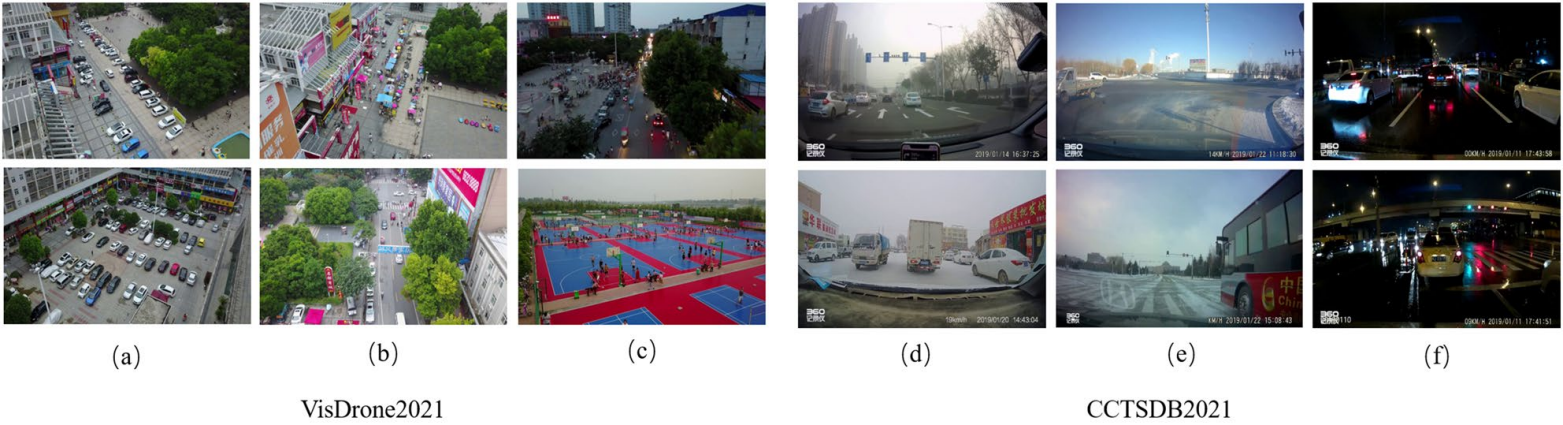
Representative image of VisDrone2021 and CCTSDB2021.

The first dataset is VisDrone^20^, which contains ten categories. The small object is 60.5%. The training set has 6471 images, the validation set has 548 images, and the test set has 3190 images. The dataset is captured by UAVs at different heights, with large differences in object scales, complex backgrounds, and variable viewpoints, which can be very different for the same object with different viewpoints. A representative picture of the dataset is shown in Fig. 8. Figure 8a shows the multi-scale object image under the dense image directly below looking down. Figure 8b shows the dense small object image in a complex background from a top-down slant perspective. Figure 8c shows the top-down view of dense small object images under different lighting conditions under a larger slant Angle (more prone to occlusion).

The second dataset is CCTSDB2021^21^, which is the authoritative traffic dataset in China. There are three common types of traffic signs. This dataset comes from the actual driving scene, and there are many cases of night light interference and bad weather interference. There are 16354 images for training and 1500 images for validation. This is shown in Fig. 8. Figure 8d represents the small object detection image with a complex background in different weather. Figure 8e represents the small object image under a simple background. Figure 8f represents the small object detection image with a dark background and different weather (the rain reflection on the road surface is easy to cause more visual errors).

**Figure 9:**
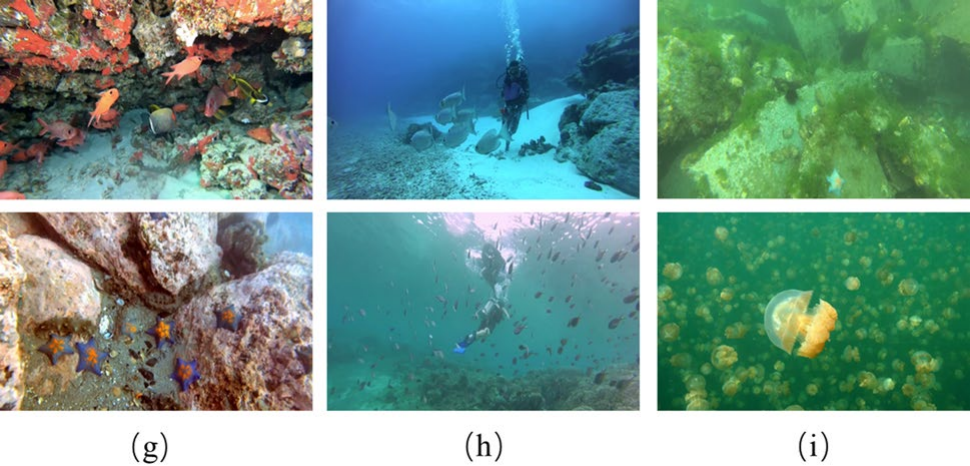
Representative image of ROUD2023.

To further validate the generalization of FocusDet for small object detection, the third dataset is the underwater small object detection dataset ROUD2023^22^. ROUD underwater object detection dataset. The dataset contains 9800 images in the training set and 4200 images in the test set. The dataset contains 10 species of marine organisms. For robotic underwater detection, dense objects present a significant problem. Moreover, different depths underwater are subject to different light conditions, and the clarity is also affected by sediment. The complex background makes this dataset a good validation of FocusDet’s performance. According to Fig. 9. Figure 9g shows an example of a complex marine object containing occlusions, small objects, various deformations, and blurred appearance Fig. 9h Example of a small object subjected to light interference. Because the ROUD dataset was captured from a variety of scenes. Artificial light, uneven illumination, and sunlight can produce light interference. A few instances of small objects with fog effects are shown in Fig. 9i. Detection of similarly sized objects in low definition is prone to false detection.

### Implementation details

FocusDet adds locally-enhanced position encoding attention, a Space-to-depth module, an enhanced feature fusion module, and SIOU-SoftNMS. All the models are implemented on PyTorch1.12.1 and trained and tested using two NVIDIA RTX3090ti GPUs. Table 1 displays the hyperparameter settings.

**Table 1:** Hyperparameter settings.

Hyperparameter name	Number
Number of epoch	200
Batch_size	16
Input size	640
Optimizer	SGD
Initial learning rate	1e-2
Momentum	0.937
Weight_decay	5e-3
Warmup_epoch	3

### Evaluation of datasets and comparative experiments

Datasets from CCTSDB and VisDrone are used for the experiments. To highlight the FocusDet’s effectiveness for detection, which is compared with the most advanced object detector. As shown in Table 2. STCF-EANet has only 81% of the parameters of ResNet18 and its GFLOP is lower than ResNet50. FocusDet is compared with twelve recently popular small object detection algorithms on the VisDrone validation dataset. Specifically, RetinaNet^23^,DMDet^24^,ClusDet^25^,GLSAN^26^, QueryDet^2^, CascadeNet^27^ use ResNet-50 as backbone. GFL V1(CEASC)^28^ and DFPN^3^ chose Modified CSP v5-M as its backbone. HRDNet^29^ uses both ResNet-18 and ResNet-101. Even though FocusDet uses lower-resolution images, it achieves the best results on the main evaluation metrics. As shown in Table 3, this result proves that FocusDet can improve efficiency.

To illustrate the benefits of the FocusDet even further, On the VisDrone test set, FocusDet is assessed once more and contrasted with SSD512^30^, FPN^31^, RetinaNet^23^, YOLOv3^11^, YOLOx^32^, and SSD512^30^. The L and S models of the original YOLOv5, YOLOv7-tiny^33^, Efficitive-Lightweight YOLO^34^, and Improved YOLOv5^35^ are compared. And evaluating FocusDet on the CCTSDB2021 test set, And it is compared with Fast-RCNN^7^, Dynamic-RCNN^36^, Sparse-RCNN^37^,SSD^30^,YOLOv5s,YOLOv7-tiny^33^, SC-YOLO^38^. Metrics including mean average precision (mAP), recall, and model precision were used to assess performance. Tables 3, 4, and 5 give the particular results.

Table 3 shows the input is configured to a resolution of 768*768 to emphasize the excellent performance of FocusDet. Even ClusDet and DMNet use ResNext - 101 the backbone of more complex, or RetinaNet ClusDet, DMNet, QueryDet, and GFL V1 using higher resolution. FocusDet scored 30.4% on the key evaluation metric mAP@.5:.95%. This performance far exceeds other advanced algorithms.

Table 4 shows that on the VisDrone test set, FocusDet achieves a mAP@.5% of 40.6%, which is an increase of 8.9% compared to YOLOv5s. Compared with Improve YOLOv5, the mAP@0.5% is increased by 2.1%. It is 6.7% higher than YOLOv5-Large and 12.8% higher than YOLOv7-tiny. mAP@.5:0.95% reaches 23.9%, which is 6.3% higher than YOLOv5s and 2.1% higher than ImproveYOLOv5.

Table 5 shows that FocusDet’s accuracy is 2.5% higher than FAST-RCNN’s when compared to the Fast RCNN model with more parameters on the CCTSDB2021 dataset. Compared with YOLOv5s, the mAP@.5% is increased by 6.2% with a similar number of parameters and only 2M more parameters. Compared with YOLOv7-tiny, the mAP@.5% is increased by 6.9% with 3M more parameters. Compared with SC-YOLO, mAP@.5% is 3.5% higher. In conclusion, FocusDet achieves the best detection accuracy with minimal parameters.

As shown in Table 6, FocusDet performance was again evaluated using ROUD2023. In comparison with many types of algorithms, FocusDet made the best of it in detection accuracy. In One-stage, FocusDet uses STCF-EANet to achieve mAP@.5:.95% 62.2% and mAP@.5%84.8% . mAP@.5:.95% outperforms FreeAnchor^39^, which ranks second in One-stage accuracy, by 7.2%. The best Multi-stage DetectoRS^40^ uses ResNet50, with mAP@.5:.95% reaching 57.8% and mAP@.5% reaching 83.6%. However, it is still worse than FocusDet and mAP@.5:.95% is 4.4% ahead of DetectoRS. The best Key-point based approach is RepPoints^41^, which uses ResNet101 as Backbone. The mAP@.5:.95% reached 55.4%. mAP@.5:.95% is 6.8% lower than FocusDet. The best Center-point based approach is Guided Anchoring^42^. Using ResNetXt101 as the Backbone, mAP@.5:.95% reaches 56.7%. FocusDet mAP@.5:.95% outperforms Guided Anchoring by 5.5%. In addition, it achieves the best results not only on mAP@.5:.95% but also on Params and GLOPS. Table 2 shows that the STCF-EANet Params used by FocusDet is only 9.27M and GLOPS is only 33.7. The results demonstrate that FocusDet can effectively detect small objects against complicated backgrounds.

**Table 2:** Comparative analysis of different backbone network structure parameters and GFLOPs.

Backbone	Param (M)	GLOPS
ResNet18	11.18	**29.78**
ResNet50	23.50	67.45
ResNet101	42.50	128.39
ResNext101_32*4d	42.13	131.48
ResNext101_64*4d	81.41	254.42
STCF-EANet(ours)	**9.27**	33.70

Significant values are in bold.

**Table 3:** Comparison of different models on VisDrone validation set.

Method	Backbone	Resolution	mAP@.5%	mAP@.75%	mAP@.5:.95%
RetinaNet^23^	ResNet-50	2400*2400	44.9	27.1	26.2
ClusDet^25^	ResNet-50	1000*600	50.6	24.4	26.7
ClusDet	ResNext-101	1000*600	**53.2**	26.4	28.4
DMNet^24^	ResNet-50	1000*600	47.6	28.9	28.2
DMNet	ResNext-101	1000*600	49.3	30.6	29.4
GLSAN^26^	ResNet-50	1000*600	51.5	22.9	25.8
HRDNet^29^	ResNet-50+ ResNet-101	2666*1600	49.3	28.2	28.3
QueryDet^2^	ResNet-50	2400*2400	48.1	28.8	28.3
GFL V1^4^	ResNet18	1333*800	50.0	27.8	28.4
GFL V1(CEASC)^28^	ResNet-18	1333*800	50.7	28.4	28.7
Cascade^27^	ResNet-50	–	47.1	29.3	28.8
DFPN^3^	Modified CSP v5-M	768*768	50.9	30.5	30.3
YOLOv8	CSPDarkNet	640*640	37.6	–	22.1
FocusDet(ours)	STCF-EANet	768*768	48.7	**35.6**	**30.4**

The bolded performance is the best one.

**Table 4:** Comparison of different models on VisDrone-test-dev set.

Model	Precision	Recall	mAP@.5%	mAP@.5:.95%
SSD512^30^	11.0	40.5	23.9	–
FPN^31^	27.3	39.7	29.2	–
RetinaNet^23^	13.8	29.9	21.2	–
YOLOx-s^32^	24.6	44.6	33.8	20.2
YOLOx-l	35.4	44.4	37.1	21.1
YOLOv3^11^	45.9	34.8	32.3	18.3
YOLOv3-spp	49.4	33.7	32.4	18.1
YOLOv5-s	43.8	34.3	31.7	17.6
YOLOv5-l	31.4	46.2	33.9	19.2
YOLOv7tiny^33^	41.2	33.7	28.8	14.5
ImproveYOLOv5^35^	36.9	**49.6**	38.5	21.8
EL-YOLO-s^34^	**54.1**	44.5	–	21.4
FocusDet(ours)	46.0	35.4	**40.6**	**23.9**

The bolded performance is the best one.

**Table 5:** Comparison of different models on the CCTSDB2021.

Model	Precision	Recall	F1	mAP@.5%	Params(M)
Fast RCNN^7^	84.4	54.9	66.5	56.5	143.7
Libra RCNN^43^	83.7	60.0	70.0	61.4	–
Dynamic RCNN^36^	87.0	58.3	69.8	60.0	–
Sparse RCNN^37^	**94.1**	52.6	67.6	59.7	–
SSD^30^	86.5	27.4	42.0	49.2	–
YOLOv3^11^	84.6	42.7	56.8	50.0	–
YOLOv4^44^	76.2	52.5	62.2	51.7	–
YOLOv7-tiny^33^	89.8	74.9	81.7	80.9	6.2
YOLOv5-s	91.2	76.8	83.3	81.6	7.2
SC-YOLO^38^	93.8	76.8	**84.5**	84.3	**6.1**
FocusDet(ours)	92.2	**76.9**	83.9	**87.8**	9.26

The bolded performance is the best one.

**Table 6:** Comparison of different models on the ROUD2023.

Method	Model	Backbone	mAP@.5:.95%	mAP@.5%	mAP@.75%	AP-s%	AP-m%	AP-l%
One-stage	SSD^30^	VGG16	43.4	73.4	45.4	11.7	31.6	48.4
	RetinaNet^23^	ResNetXt101	50.7	79.3	54.5	14.3	39.2	56.1
	FreeAnthor^39^	ResNetXt101	55.0	82.4	59.8	17.0	42.9	60.7
	NAS-FPN^13^	ResNet50	51.4	78.9	55.2	14.4	38.3	56.7
	ATSS^40^	ResNet101	52.9	80.3	56.9	16.4	41.1	58.6
	YOLOF^45^	ResNet50	50.1	80.0	53.8	11.2	37.4	55.9
	FocusDet(ours)	STCF-EANet	**62.2**	**84.8**	**64.3**	17.1	**44.2**	**63.4**
Two-stage	Faster R-CNN^8^	ResNetXt101	52.8	81.8	57.5	17.2	40.9	58.2
	Cascade R-CNN^27^	ResNetXt101	54.8	81.1	59.7	16.8	42.2	60.6
	Dynamic R-CNN^36^	ResNet50	54.4	81.3	60.3	17.1	42.8	60.0
	DetectoRS^40^	ResNet50	57.8	83.6	63.6	**20.4**	45.0	63.7
	Libra R-CNN^43^	ResNetXt101	54.8	82.8	60.5	16.5	43.1	60.6
	ThunderNet^46^	ShuffleNetV2	41.7	67.9	44.6	8.8	25.6	46.7
Key-point based	Grid R-CNN^47^	ResNetXt101	53.7	81.1	58.4	17.7	41.2	59.1
	RepPoints^41^	ResNet101	55.4	83.7	60.4	17.7	43.3	60.8
	CornerNet^15^	HourglassNet	41.9	60.3	43.7	9.5	33.2	43.7
Center-point based	FCOS^48^	ResNetXt101	50.7	79.5	50.4	18.0	40.0	56.2
	FoveaBox^49^	ResNet101	52.1	81.4	56.0	15.1	40.5	57.5
	FSAF^50^	ResNetXt101	48.7	78.5	51.2	15.7	38.0	53.9
	Guided Anchoring^42^	ResNetXt101	56.7	84.2	62.0	18.1	44.0	62.6

The bolded performance is the best one.

### Ablation experiments

Tables 7, and 8 show that each additional improvement proposal received positive feedback. On the VisDrone dataset, Table 7 demonstrates that the original model’s mAP@.5% is 33.6%. After using STCF-EANet, the mAP@.5% is improved by 2.2%. After using Bottom Focus-PAN, the mAP@.5% is increased to 41.7%, an increase of 5.9%. After replacing the NMS of the original network with SIOU-SoftNMS, it is improved to 46.7%, an increase of 5 On the CCTSDB2021 dataset, Table 8 demonstrates that the original model’s mAP@.5:.95% is 54.6%. After using STCF-EANet, the mAP@.5:.95% is improved by 1.9%. After using Bottom Focus-PAN on this basis, the mAP@.5:.95% is improved to 57.3%. After replacing the NMS of the original network with SIOU-SoftNMS, it is improved to 63.2%, an increase of 5.9%. The above experiments illustrate the good feasibility of using solutions STCF-EANet, Bottom Focus-PAN, and SIOU-SoftNMS for small object detection.

**Table 7:** Ablation experiments of VisDrone (val).

Method	STCF-EANet	Bottom focus-PAN	SIOU-SoftNMS	mAP@.5%
01	–	–	–	33.6
02	$\checkmark$	–	–	35.8
03	$\checkmark$	$\checkmark$	–	41.7
04	$\checkmark$	$\checkmark$	$\checkmark$	**46.7**

Significant values are in bold.

**Table 8:** Ablation experiments for CCTSDB2021.

Method	STCF-EANet	Bottom focus-PAN	SIOU-SoftNMS	mAP@.5%	mAP@.5:.95%
01	–	–	–	81.6	54.6
02	$\checkmark$	–	–	83.9	56.5
03	$\checkmark$	$\checkmark$	–	84.0	57.3
04	$\checkmark$	$\checkmark$	$\checkmark$	**87.8**	**63.2**

Significant values are in bold.

### Visual comparisons

**Figure 10:**
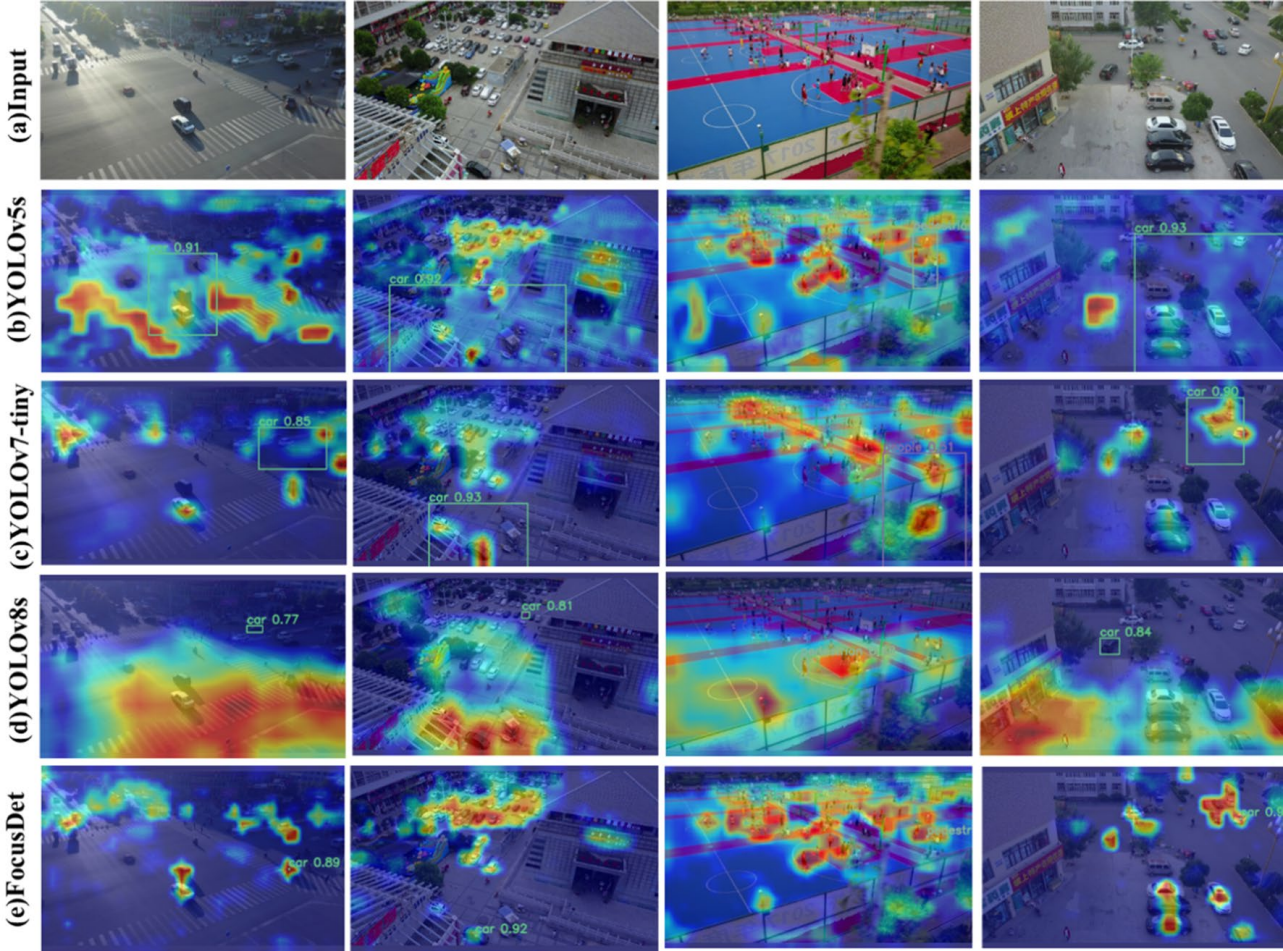
Visual comparison of receptive fields.

The superior performance of FocusDet was compared using the Grad-CAM visualization approach. As shown in Fig. 10, compared with YOLOv5s, YOLOv7-tiny, and YOLOv8, FoucsDet benefits from STCF-EANet, making the attention field of the image more accurate and focused. Small objects can be accurately captured and are well avoided for irrelevant semantic information.

**Figure 11:**
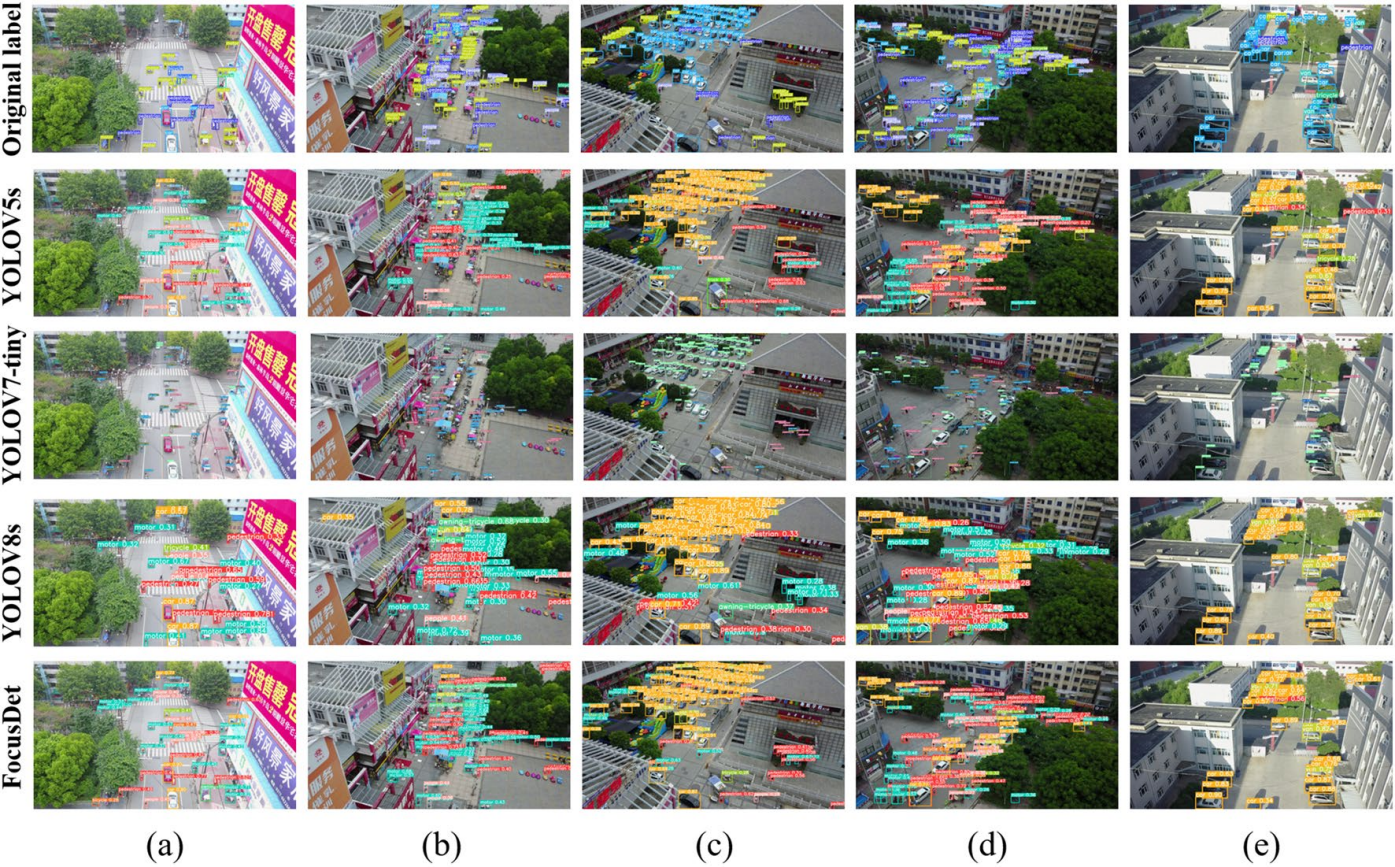
Comparison of detection effect under complex background.

**Figure 12:**
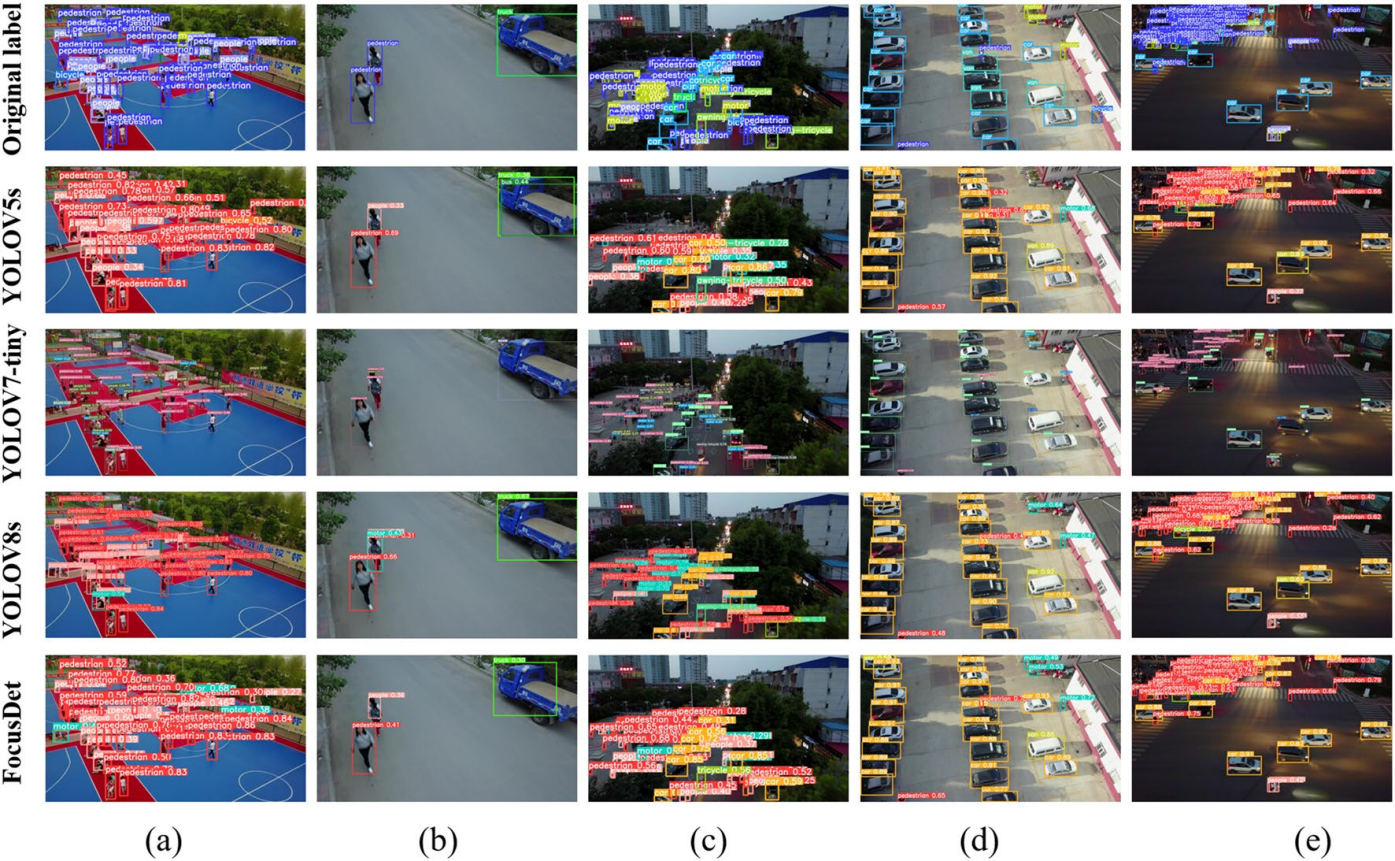
Comparison of detection effects under different light backgrounds.

Figures 11 and 12 shows a comparison using a typical picture of VisDrone, a viewpoint of more common application scenarios. Compared with YOLOv5s, YOLOv7-tiny, and YOLOv8 networks, FocusDet is more adept at identifying small objects in a variety of scenarios. In Fig. 11, there are some classical views. The view contains many dense small objects. These small objects are occluded in different views. This brings great difficulties to the detection of YOLOv5s, YOLOv7-tiny, and YOLOv8. The figure displays the recognition range of YOLOv5s is small, and serious detection omissions will occur for small object objects near the boundary of the visual field. YOLOv7-tiny is slightly better than YOLOv5s in detecting such images, but its recognition accuracy is lower. For example, in the motor recognition in image (b) in Fig. 11, the detection omission problem also occurs. YOLOv8 also has the problem of missing detection. FocusDet performs extremely well on the missed detection problem that arises in the detection of complex tasks such as occlusion.

Figure 12 shows the detection comparison under different light backgrounds. The environmental background of images (b) and (d) in Fig. 12b is relatively simple, and the object occlusion and object aggregation are not serious. Such routine checks are easily done by FocusDet. YOLOv5s, YOLOv7-tiny, and YOLOv8 do not show good detection results. YOLOv5s shows duplicate detection and false detection, YOLOv7-tiny shows duplicate detection, and YOLOv8 shows false detection. In (a), (c), and (e), the results of dense small object detection under different light backgrounds are shown. YOLOv7-tiny incorrectly identifies people as bicycles in a crowd. YOLOv5s focuses on the dense part of the picture when detecting dense images. However, it ignores the detection of boundaries and sparse parts. YOLOv8 performs better under daily light conditions, but duplicate detection occurs under the influence of night light. Under different lighting backgrounds, FocusDet can detect small boundary objects well. It effectively solves the miss-detection problem.

**Figure 13:**
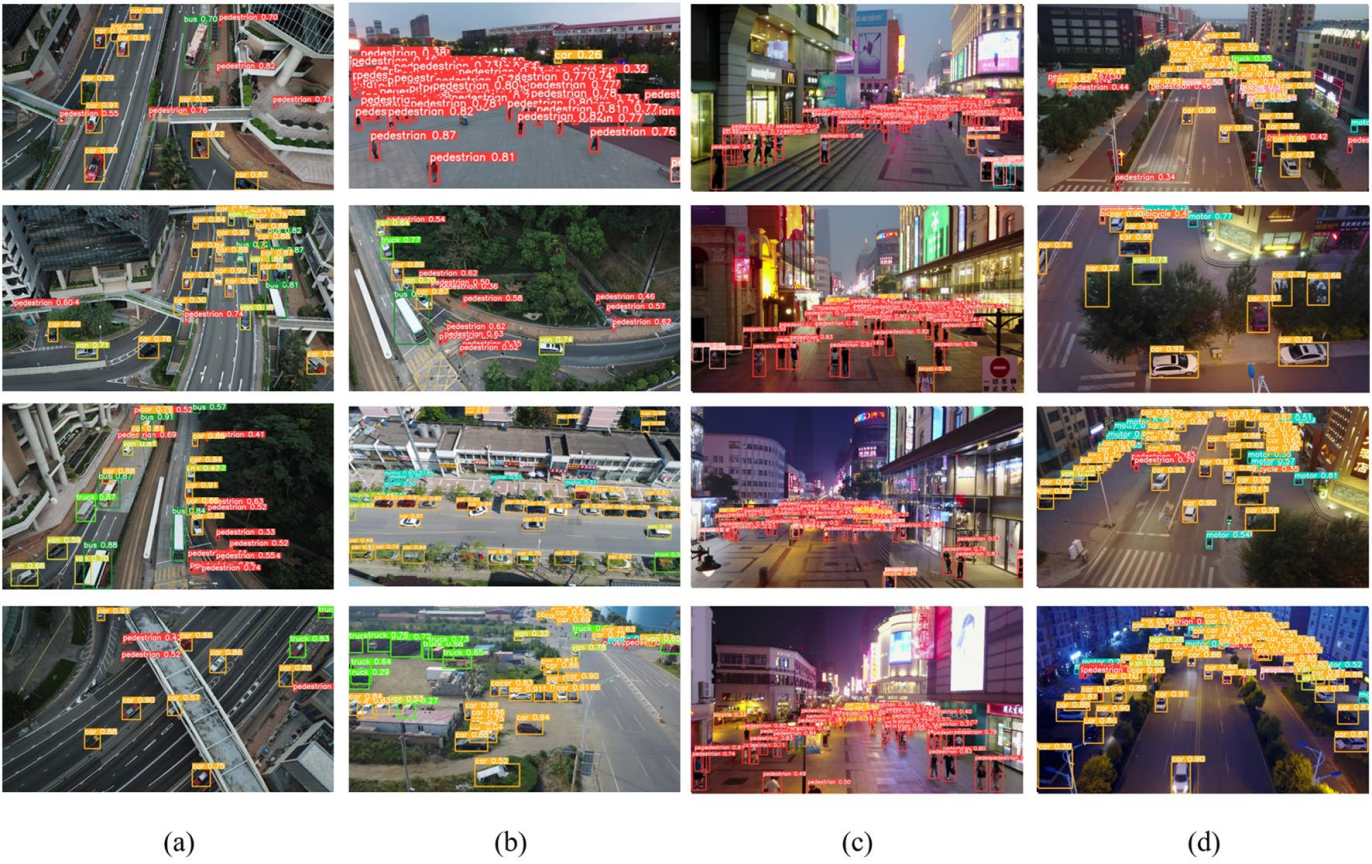
Detection effect display of FocusDet.

The selection of challenging images demonstrates the superior detection performance of FocusDet. Cars in complex backgrounds in Fig. 13a are accurately recognized by FocusDet. Without the interference of a rectangular green background and tree branch occlusion, Fig. 13b shows that FocusDet can still accurately detect small objects. The dense object detection under oblique viewing angles with different lighting conditions at night in Fig. 13c,d works well. In conclusion, in the face of the challenges of complex background interference, small objects, and large object size span, the FocusDet model can accurately locate and identify objects.

## Conclusion

This paper analyzes the shortcomings of general object detectors in small object scenarios and proposes solutions based on the difficulties. Small object detection mainly contains three difficulties: small object size, dense objects, and sophisticated background noise. This leads to the general object detector can not handle small objects well. So the small object detector FocusDet is proposed to solve the above three difficulties.STCF-EANet is designed to extract small object features more accurately. Bottom Focus-PAN complements small object feature details by feature fusion.SIOU-SoftNMS is used to solve the omission phenomenon under dense objects.

Based on the above methods.On the visdrone dataset, mAP@.5:95% achieves 23.9%, an increase of 6.3% compared to the baseline. On the CCTSDB2021 dataset,mAP@.5% reaches 87.8%, which is 6.2% higher than the baseline. Compared with a variety of algorithms on the ROUD2023 dataset, FocusDet has the best effect and mAP@.5:95% reaches 62.2%. The quantitative evaluation results show that FocusDet can achieve the best small object detection performance while maintaining a small number of parameters. The qualitative evaluation results show that FocusDet can effectively utilize the features of small objects in various scenarios, and overcome the problems of false detection, missed detection, and repeated detection. FocusDet can handle detection in small object scenes well with good generalization ability. It can achieve good results in various scenes of traffic, UAV, and underwater small object detection. Faced with small object detection in complex scenes, FocusDet can accurately locate and identify the object. It promotes the progress of small object detection algorithms.

To further study this topic in depth, the future research mainly focuses on two aspects: (1) Improve the algorithm to improve the phenomenon that similar objects are prone to false detection. (2) To enhance the network structure, become knowledgeable about the newest object-detecting technologies. Maintain high detection accuracy while reducing model complexity.

## Data Availability

The datasets used in this study are publicly available. The VisDrone dataset is available on the official website: https://github.com/VisDrone. The CCTSDB dataset is available on the official website: https://github.com/csust7zhangjm/CCTSDB. ROUD is a datasets created by publicly published papers: Fu, C. et al. Rethinking general underwater object detection: Datasets, challenges, and solutions. Neurocomputing 517, 243-256 (2023).
